# Effects of Gamma-Ray Irradiation of Bacteria Colonies in Animal Feeds and on Growth and Gut Health of Weaning Piglets

**DOI:** 10.3390/ani13213416

**Published:** 2023-11-03

**Authors:** Hao Wei, Min Yang, Xuemei Jiang, Lun Hua, Chao Jin, De Wu, Yan Wang, Yong Zhuo

**Affiliations:** 1 Animal Nutrition Institute, Key Laboratory for Animal Disease Resistant Nutrition of the Ministry of Education, Sichuan Agricultural University, Chengdu 611130, China; weih2008@126.com (H.W.); yang040101@yeah.net (M.Y.); 71310@sicau.edu.cn (X.J.); hualun@sicau.edu.cn (L.H.); jinchao@sicau.edu.cn (C.J.); wude@sicau.edu.cn (D.W.); 2 Pet Nutrition and Health Research Center, Chengdu Agricultural College, Chengdu 611130, China; 3 College of Animal Science and Technology, Sichuan Agricultural University, Chengdu 611130, China; wangyan519@sicau.edu.cn

**Keywords:** γ-ray irradiation, *E. coli*, feed bacteria, fishmeal, piglets

## Abstract

**Simple Summary:**

Animal feeds contain a substantial number and diversity of microorganisms due to bacterial contamination during processing, transportation, and storage, and some of them have pathogenic potential. To test the effect of gamma (γ)-ray irradiation on the elimination of bacteria in different feeds, we treated fishmeal, feather meal, meat meal, soybean meal, and vitamin complexes with graded doses of γ-ray irradiation. We found that a dose of 6–9 kGy was sufficient to eliminate most of the bacteria in the investigated feed. We also found that fishmeal with a high bacterial load and *Escherichia coli* (*E. coli*) contamination induced an increased diarrhea index and impaired the growth performance of weaning piglets but that these effects were reduced by γ-ray irradiation treatment at 6 kGy. These findings demonstrate that γ-ray irradiation can be used to treat feeds with biosafety concerns.

**Abstract:**

Animal feeds contain a substantial number and diversity of microorganisms, and some of them have pathogenic potential. The objectives of this study were to investigate the effects of different doses of gamma (γ)-ray irradiation on the bacteria count in different types of feed and then to test the effect of γ-ray-irradiation-treated fishmeal on the gut health and growth performance of weaning piglets. In trial 1, three fishmeal samples, two feather meal samples, three meat meal samples, three soybean meal samples, and three vitamin complexes were treated with γ-ray irradiation doses of 0, 3, 6, or 9 kGy. The 6 and 9 kGy doses eliminated most of the bacteria in the feed but also resulted in a loss of vitamin C and B1. In trial 2, 96 weaning piglets were fed one of the following three diets with eight replicates (pens) per group over a 14-day period: (1) the control diet—the basal diet supplemented with 6% fishmeal with a low bacteria count (40 CFU/g) and no *E. coli*; (2) the fishmeal-contaminated diet (FM-contaminated) diet—the basal diet supplemented with 6% fishmeal with a high bacteria count (91,500 CFU/g) and *E. coli* contamination; and (3) the irradiated fishmeal (irradiated FM) diet—the basal diet supplemented with γ-ray-irradiation-treated *E. coli*-contaminated fishmeal. The piglets that received the FM-contaminated diet had significantly lower average daily gain and a greater diarrhea index compared to those fed the control diet, whereas γ-ray irradiation treatment abrogated the negative effect of the *E. coli*-contaminated fishmeal. Collectively, γ-ray irradiation at a dose of 6–9 kGy was sufficient to eliminate the microorganisms in the feed, thereby benefitting the growth performance and gut health of the weaning piglets.

## 1. Introduction

The ban on antibiotics use in animal feed has led to health problems in swine production that must be overcome to avoid economic losses. In particular, the immature gastrointestinal tract of weaning piglets predisposes them to susceptibility to disease [1]. Environmental factors, such as bacteria in feed, are among the primary exogenous factors that negatively impact the health of piglets [2]. Indeed, numerous bacteria are present in animal feed, which is vulnerable to the introduction of bacteria due to the inclusion of contaminated raw ingredients and/or because of contamination during transportation and feed processing [3,4]. The use of protein feed of animal origin, such as fish, meat, and bone meal and animal plasma protein, is of great concern because it is vulnerable to contamination by pathogenic bacteria such as *E. coli* and *Salmonella* [3,5,6]. For example, the global epidemic of African swine fever virus has raised concern among swine producers about the use of porcine plasma protein in feed [5].

There are several ways of reducing the bacteria counts in animal feed, such as heat treatment [7], acidification [8], and ultraviolet irradiation [9]. However, these methods cannot eliminate bacteria completely and sometimes negatively impact nutritional value [10]. An alternative treatment is irradiation sterilization, in which electromagnetic waves generated by ionizing radiation, such as electron beams, X-rays, and gamma (γ)-rays, are used [11,12,13]. Irradiation treatment is a process that involves energic, physical, and chemical changes but does not cause the irradiated substance to produce emissivity. Irradiation causes energy output, and the irradiated material absorbs that energy. It is usually expressed as gray, 1 gray, or 1 kg of irradiated material absorbed per 1 joule of energy (1 Gy = 1 J kg^−1^), which is similar in nature to the heat generated by heat (infrared) or microwave energy [14]. Recently, Li et al. reported that γ-ray irradiation is an effective method for reducing bacterial counts in feed and food [15]. They found that it can even modify the structure of dietary polysaccharides by cleaving their glycosidic bonds and exposing more functional groups with increased mobility [15]. However, the optimal level of γ-ray irradiation to apply to lower the bacterial counts in feed remains unclear. Additionally, the effect of γ-ray irradiation treatment on the feeding value of contaminated feed material in the animals consuming it is unknown. 

To address these issues, we conducted two trials. In trial 1, we tested the effects of different levels of γ-ray irradiation treatment on 14 types of feed to identify the optimal irradiation dosage. In trial 2, we treated contaminated fishmeal with γ-ray irradiation to investigate the role of bacterial counts in the feed on the growth performance of weaning piglets.

## 2. Materials and Methods

### 2.1. Study Approval

The experimental procedures were performed in accordance with the Institutional Animal Care and Research Committee of Sichuan Agricultural University, Chengdu, China, which approved this study (protocol number: SICAU-2019-0021).

### 2.2. Trial 1: Feed Samples and γ-Ray Irradiation Treatment

The 14 feed raw materials tested in this study consisted of three fishmeal samples, two feather meal samples, three meat meal samples, three soybean meal samples, and three vitamin premix products obtained from different manufacturers. These feed samples were treated with 0, 3, 6, or 9 kGy of γ-ray irradiation, and each feed stuff/dosage combination had three replicates.

Samples were tightly sealed in vacuum packages to prevent contamination with airborne microorganisms after irradiation. All experimental steps in the γ-ray irradiation process were carried out at Sichuan Jinnuo Irradiation Technology Co., Ltd. (Meishan, China). The irradiation process conducted complies with the basic provisions issued by Decree of the National Council of the People’s Republic of China (No. 44) on 24 October 1989 as well as the relevant national standard “Regulations on Radiation Protection of ^60^Co Irradiation Devices for Irradiation Processing” (GB10252-88) [16].

The bacterial count in the sample was determined following the Chinese standard GB 4789.2-2016 [17]. In summary, 25 g of feed samples was diffused into 225 mL of potassium dihydrogen phosphate solution (0.068 g/mL, pH = 7.2), and a series of 10-fold diluted samples were prepared. Liquid samples were then plated onto agar medium to observe growth at 36 °C (±1) for 48 h. Bacterial counts were recorded as colony-forming units (CFU), and the results were expressed as the total number of aerobic plate counts per gram of feed (CFU/g). 

Total volatile basic nitrogen (TVB-N) was measured using the micro-diffusion method, following the Chinese standard GB 5009.228-2016 [18]. Briefly, 5g portions of finely ground fish meal samples were mixed with 50 mL of distilled water, followed by homogenization and filtration. Then, 10 mL of the filtrate was suspended with 10 mL of distilled water and 5 mL of MgO. This mixture underwent 5 min of steam distillation. Subsequently, 10 mL of boric acid solution (20 g/L) was added and mixed with 5 drops of indicator solution (methyl red: bromocresol green = 1:2, *v/v*). The solution was then titrated with 0.01 M HCL, and the results were expressed as the total milligrams of TVB-N per 100 g of feed (mg/100 g).

The content of vitamins A and E in the feeds was determined using high-performance liquid chromatography in accordance with the Chinese standard GB/T 5009.82-2003 [19], which has been stated previously [20]. 

The vitamin C content in the feeds was measured using the 2,6-dichloroindophenol titration method, following the Chinese standard GB 5009.86-2016 [21]. In summary, finely ground feed samples were homogenized with metaphosphoric acid solution (20 g/L) in a 1:1 ratio. Subsequently, 20 g portions of the homogenized samples were transferred to volumetric flasks to create a 100 mL volume with metaphosphoric acid solution, and they were then used for titration with calibrated 2,6-dichloroindophenol solution.

The vitamin B1 content in the feeds was determined using high-performance liquid chromatography, following the national standard GB 5009.84-2016 [22]. In brief, the feed samples were hydrolyzed, neutralized, and enzymatically hydrolyzed at a constant temperature in a dilute hydrochloric acid medium. The hydrolysate content was derived with alkaline potassium ferricyanide solution for n-butanol extraction. The extracts were then processed through C18 reversed-phase column separation and detected using a high-performance liquid chromatography fluorescence detector.

### 2.3. Trial 2: Animals, Diets, and Experimental Design

Ninety-six crossed Duroc × (Landrace × Yorkshire) piglets that started being weaned at 28 days old (±1 day) and had a body weight of 6.98 kg (±0.12 kg) were randomly allocated into three groups for a 14-day feeding experiment. Each of the three dietary treatments was applied to eight pens, and each pen contained four piglets (two barrows and two gilts). The dietary formulations (Table 1) containing 6% fishmeal from different sources were as follows: (1) control diet—the basal diet supplemented with 6% fishmeal with low CFU and no *E. coli*; (2) fishmeal-contaminated (FM-contaminated) diet—the basal diet supplemented with 6% fishmeal with high CFU and contaminated with *E. coli*; (3) irradiated fishmeal (irradiated FM) diet—the basal diet supplemented with γ-ray-irradiation-treated *E. coli*-contaminated fishmeal. The basal diet was treated with γ-ray irradiation at a dose of 6 kGy.

After being weaned at the age of 25 days, the piglets were allowed to adapt to the environment of the nursing facility for 3 days, and then the experiments began on day 28. Each pen was equipped with two nipples supplying water and one self-feeder. Piglets were provided with feed and water ad libitum, and piglets were fed the diets four to six times per day. The feeding conditions were examined every 2 h to ensure that the feed was provided freely but without waste. The temperature and humidity were maintained at 24–26 °C and 50–70%, respectively. Table 2 shows the nutrient content of each diet. Diets were formulated in mashed form and prepared such that they met or exceeded the nutrient requirements as recommended by the NRC [23].

#### 2.3.1. Measurement of Growth Performance and Diarrhea

The body weights of the piglets were recorded on days 1, 7, and 14 of the experiment, using pens as the experimental units, and the average daily gain (ADG) was calculated. The consumption of feed was recorded daily to calculate the average daily feed intake (ADFI). The feed conversion ratio (FCR; feed consumed/weight gain) was calculated in accordance with the ADFI and ADG. The diarrhea index was measured based on the appearance of feces and monitored at 9:30, 14:30, and 20:00 daily in accordance with the previously reported description [24]. Specifically, a score of 0 indicated firm or granular feces; 1 indicated soft but well-formed feces; 2 indicated thick, unseparated, unformed liquid feces; and 3 indicated unshaped, separated liquid feces. 

#### 2.3.2. Measurement of the Number of *E. coli* Colonies in Feces

On day 14 of the trial, feces were collected to measure the number of *E. coli* present, following a method described in a previous study [25]. In brief, the feces collected from the four piglets in each pen were equally pooled together to constitute one sample. Fecal samples were diluted in a gradient manner (10^−1^–10^−7^) with sterilized saline solution and then plated onto MacConkey agar plates. The number of viable *E. coli* bacteria in each sample was calculated using the following equation: *E. coli* colonies (CFU)/g = number of *E. coli* colonies on the plate × dilution ratio of fecal sample/fecal weight [25].

#### 2.3.3. Collection and Measurement of Serum Parameters

Blood samples were collected from the jugular vein of one piglet (barrow) in each pen at 08:00 before the morning meal on day 14 of the experiment and placed in heparinized vacuum tubes. Plasma was harvested by centrifuging the blood samples at 3000× *g* for 15 min at 4 °C, and the plasma samples were stored at −20 °C. Subsequently, they were used to measure the levels of serum parameters of immunity-related cytokines. C-reactive protein was measured using commercial enzyme-linked immunosorbent assay (ELISA) kits purchased from Cusabio Biotech (catalog CSB-E08163p, Wuhan, China) with minimum detection of 0.16 ng/mL. Interleukin (IL)-1β (catalog CSB-E06782p) and IL-6 (catalog CSB-E06786p) levels were measured using ELISA kits from Cusabio Biotech (Wuhan, China), and their minimum detection values were 0.975 pg/mL and 0.31 pg/mL, respectively.

### 2.4. Statistical Analysis

Experimental data were checked for homogeneity of variances and normal distribution of the residuals before using parametric analyses. For the data from trial 1, there were three replicate samples for each dosage of γ-ray irradiation treatment. For data from trial 2, the pens were considered to be replicates, and the statistical analyses were performed using the mixed procedure of SAS 9.4 (SAS Institute Inc., Cary, NC, USA) in a completely randomized design. Multiple comparisons between every two groups were performed using Tukey’ test. Differences were considered to be significantly different at *p* < 0.05. 

## 3. Results

### 3.1. Trial 1: The Effect of γ-Ray Irradiation on the Bacteria Counts in Different Feeds

#### 3.1.1. Fishmeal Samples

**Figure 1:**
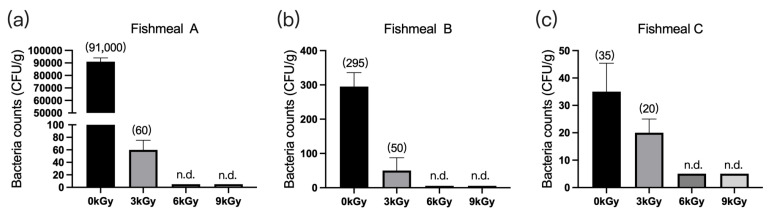
Effect of γ-ray irradiation doses on the bacterial counts of fishmeal samples. (**a**–**c**) The bacterial counts of fishmeal samples A, B, and C at γ-ray irradiation doses of 0, 3, 6, and 9 kGy. Values are means ± S.E.; *n* = 3 per sample. N.d., not detected or below detection limits. The bacteria count in the column for n.d. groups was set as 5 CFU/g. The numbers in brackets denote the average values of the bacteria counts in each sample.

The quantities of microbes in the three fishmeal samples were 91,000 CFU/g (Figure 1a), 295 CFU/g (Figure 1b), and 35 CFU/g (Figure 1c), respectively, before γ-ray irradiation. At doses of 6 and 9 kGy, the quantities of microbes per gram of feed were reduced to below detection (Figure 1a–c).

#### 3.1.2. Feather Meal Samples

**Figure 2:**
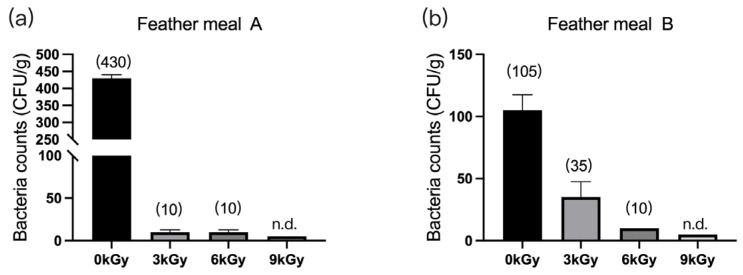
Effect of γ-ray irradiation doses on the bacterial counts of feather meals. The bacterial counts of feather meal samples A (**a**) and B (**b**) at γ-ray irradiation doses of 0, 3, 6, and 9 kGy. Values are means ± S.E., and *n* = 3 per sample. n.d., not detected or below detection limits. The bacteria count in the column for n.d. groups was set as 5 CFU/g. The numbers in brackets denote the average values of the bacteria counts in each sample.

The quantities of microbes in the two feather meal samples were 430 CFU/g (Figure 2a) and 105 CFU/g (Figure 2b), respectively, before γ-ray irradiation. After different doses of γ-ray irradiation, the quantities of microbes per gram of feed were significantly reduced, and they were below detection at a dosage of 9 kGy.

#### 3.1.3. Meat Meals

**Figure 3:**
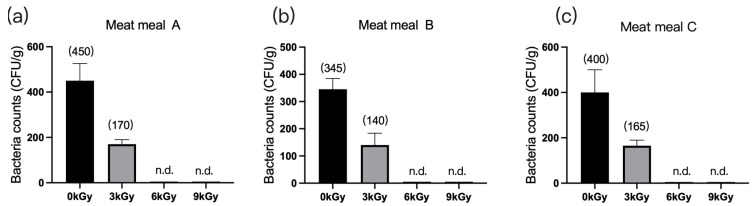
Effect of γ-ray irradiation doses on the bacterial counts of meat meal samples. (**a**–**c**). The bacterial counts of meat meal samples A, B, and C at γ-ray irradiation doses of 0, 3, 6, and 9 kGy. Values are means ± S.E., and *n* = 3 per sample. n.d., not detected or below detection limits. The bacteria count in the column for n.d. groups was set as 5 CFU/g. The numbers in brackets denote the average values of the bacteria counts in each sample.

The quantities of microbes in the three meat meal samples were 450 CFU/g (Figure 3a), 345 CFU/g (Figure 3b), and 400 CFU/g (Figure 3c), respectively, before γ-ray irradiation. At dosages of 6 and 9 kGy, the quantities of microbes per gram of feed were reduced to below detection (Figure 3a–c). 

#### 3.1.4. Soybean Meals

**Figure 4:**
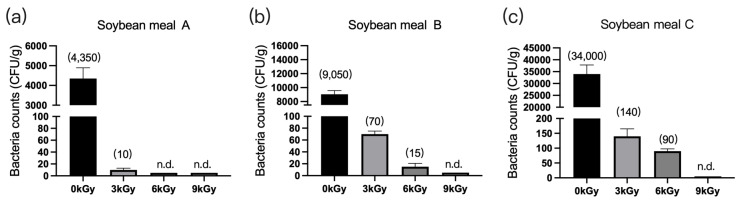
Effect of γ-ray irradiation dosage on the bacterial counts of soybean meals. (**a**–**c**) The bacterial counts of soybean meal samples A, B, and C at γ-ray irradiation doses of 0, 3, 6, and 9 kGy. Values are means ± S.E., and *n* = 3 per sample. n.d., not detected or below detection limits. The bacteria count in the column for n.d. groups were set as 5 CFU/g. The numbers in brackets denote the average values of the bacteria counts in each sample.

The microbial counts in the three soybean meal samples were 4350 CFU/g (Figure 4a), 9050 CFU/g (Figure 4b), and 34,000 CFU/g (Figure 4c), respectively, before γ-ray irradiation. At a dose of 9 kGy, the quantities of microbes per gram of feed were reduced to below detection (Figure 4a–c). 

#### 3.1.5. Vitamin Complexes 

**Figure 5:**
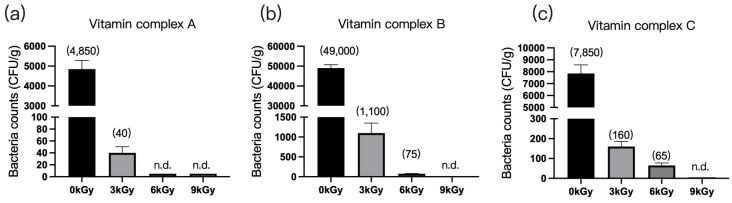
Effect of γ-ray irradiation dosage on the bacterial counts of vitamin complex. (**a**–**c**) The bacterial counts of vitamin complex samples A, B, and C at γ-ray irradiation dosages of 0, 3, 6, and 9 kGy. Values are means ± S.E., and *n* = 3 per sample. n.d., not detected or below detection limits. The bacteria counts of the columns for n.d. groups were set as 5 CFU/g. The numbers in the brackets denote average value of the bacteria counts in each sample.

The quantities of microbes in the three vitamin complex samples were 4850 CFU/g (Figure 5a), 4900 CFU/g (Figure 5b), and 7850 CFU/g (Figure 5c), respectively, before γ-ray irradiation. At a dosage of 9 kGy, the quantities of microbes per gram of feed were reduced to below detection (Figure 5a–c). 

#### 3.1.6. Effect of γ-Ray Irradiation on Amino Acid and Vitamin Profiles 

We also evaluated the effect of γ-ray irradiation on the amino acid profiles of fishmeal samples A, B, and C (Table 3). None of the amino acids were affected by different doses of γ-ray irradiation.

Additionally, we assessed the effect of γ-ray irradiation on the profiles of vitamins A, E, B1, and C in complex samples A, B, and C (Table 4). In all of the complex samples, the proportions of vitamins A and E were not affected by irradiation, and vitamin B1 content decreased as the γ-ray irradiation dose increased. The vitamin C content in complex samples A and B also decreased with an increasing γ-ray irradiation dose.

### 3.2. Trial 2: Effect of γ-Ray-Irradiation-Treated Fishmeal on the Growth Performance of Weaning Piglets

#### 3.2.1. Bacterial Counts in Feeds

The bacteria counts in fishmeal samples C and A were 40 and 91,500 CFU/g, respectively (Table 5). We detected the presence of viable *E. coli* in fishmeal sample A, and the TVB-N was 235 mg/100 g. After γ-ray irradiation, viable *E. coli* were no longer detectable, and TVB-N was reduced to 183 mg/100 g. Additionally, the bacteria count of the basal diet was below detection after γ-ray irradiation. 

#### 3.2.2. Growth Performance

Dietary supplementation with contaminated fishmeal resulted in significantly lower ADG than the other two groups during days 1–7, 8–14, and 1–14 of the experiment (*p* < 0.05). Bodyweight, ADFI, and FCR were not affected by the dietary treatment (*p* > 0.05) (Table 6). 

#### 3.2.3. Diarrhea Index and Fecal *E. coli* Counts

The piglets fed the FM-contaminated diet had greater diarrhea index values during experimental days 1–7, 8–14, and 1–14 than those fed the control diet. The piglets that received the irradiated FM diet had a dramatically reduced diarrhea index compared to the piglets fed the FM-contaminated diet (Table 7). 

**Figure 6:**
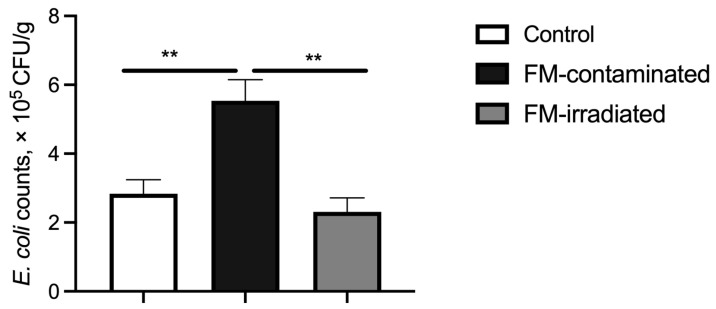
Effect of γ-ray irradiated fishmeal on the fecal *E. coli* counts of weaning piglets. The FM-contaminated diet was supplemented with 6% FM with high bacteria count and contaminated with *E. coli*. The irradiated FM diet was supplemented with 6% contaminated FM with γ-ray irradiation at a dose of 6kGy. ** denotes statistical significance at *p* < 0.01.

The fecal *E. coli* counts of the piglets fed the FM-contaminated diet were greater than those of the piglets fed the control diet. However, the fecal *E. coli* counts of the piglets fed the irradiated FM diet were lower than those of the piglets fed the FM-contaminated diet and similar to those of the piglets fed the control diet (Figure 6). 

**Figure 7:**
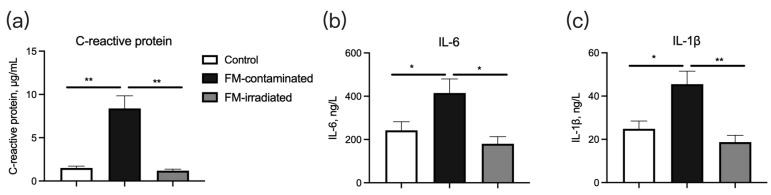
Effect of γ-ray irradiated fishmeal on the serum concentrations of immune-related cytokines in weaning piglets. The FM-contaminated diet was supplemented with 6% FM with high bacteria count and contaminated with *E. coli*. The irradiated FM diet was supplemented with 6% contaminated FM with γ-ray irradiation at a dose of 6kGy. * denotes *p* < 0.05, and ** denotes *p* < 0.01.

The serum concentrations of C-reactive protein (Figure 7a), IL-6 (Figure 7b), and IL-1β (Figure 7c) of the piglets fed the FM-contaminated diet were greater than those of the piglets fed the control diet. However, their concentrations in the piglets fed the irradiated FM diet were similar to those in the piglets fed the control diet (Figure 7a–c). 

## 4. Discussion

The gut of an animal is in direct contact with microorganisms in a feed; thus, the number and diversity of microorganisms in a feed is directly related to intestinal health and the digestion and absorption of nutrients. This is particularly true when immature piglets encounter pathogenetic microbiota such as *E. coli* [26]. In the present study, we found that fishmeal contaminated with *E. coli*. and a high bacteria load impaired the growth performance of piglets. However, γ-ray irradiation at a dose of 6 kGy effectively eliminated most of the bacteria in the feed and abrogated the negative effects of bacterial contamination on the growth performance of piglets. 

In the present study, the bacteria counts in the feeds from different sources were quite variable. The number of microbial colonies in fishmeal A was as high as 91,000 CFU/g, whereas fishmeal samples B and C contained only 295 and 35 CFU/g, respectively. The number of microbial colonies in soybean meal sample C was as high as 34,000 CFU/g, but that of soybean meal A was only 4350 CFU/g. These results indicate that fishmeal and soybean meal from different sources differ greatly in microbial counts due to the effects of processing technology, storage time, storage conditions, packaging, transportation routes, and other factors [27,28]. Fishmeal sample A also had greater TVB-N content, which consists of alkaline nitrogenous substances such as ammonia and amines that are produced via the microbial decomposition of protein, suggesting that the microbes were active during processing and storage [29]. In particular, we detected the presence of *E. coli* in the fishmeal samples. *E. coli* is commonly found in the lower digestive tract of warm-blooded organisms [30]. Most strains of *E. coli* are not harmful, but some can cause severe food poisoning. For example, Shiga-toxin-producing *E. coli* is a bacterium that can be found in raw or undercooked ground meat products. It can cause serious foodborne illness with symptoms such as abdominal cramps and diarrhea, with the latter sometimes progressing to bloody diarrhea (hemorrhagic colitis) [31]. In swine production, *E. coli* is one of the leading causes of diarrhea among newborn and weaning piglets, as it impairs the integrity of the intestinal mucosal barrier [30,32,33]. 

At present, biosafety is the primary problem that plagues the application of protein feedstuffs of animal origin, such as porcine plasma protein, fish meal, meat meal, and feather meal, in livestock production, mainly because the pathogenic microorganisms in feed raw materials cannot be killed using the conventional processing techniques [27,28,34]. γ-ray irradiation has been widely used to reduce the number of bacteria in food [35], but the appropriate treatment dose is not clear, and little is known about the effect of the γ-ray irradiation treatment of feed on weaning piglets. Therefore, the purpose of our study was to assess the effect of different doses of γ-ray irradiation on the of bacteria in feed. 

We found that γ-ray irradiation at doses of 3–9 kGy was quite effective in eliminating bacteria in the feed. Most of the feed bacteria were below the detection level at a dose of 6 kGy, and no bacteria were detectable in all of the feedstuffs treated with 9 kGy. We also found that the optimal dose of γ-ray irradiation varied for different feedstuffs. For fish meal and meat meal, all of the microorganisms were entirely eliminated at 6 kGy, but microbes were still detectable in feather meal and soybean meal at this dose, although the counts were quite low. One possible explanation for the observed differences is that the feeds differ in their bulk density. Meat meal and fishmeal are fluffy and have low bulk density, allowing γ-rays to easily penetrate entire samples of these types of meal. In contrast, soybean meal, feather powder, and other feeds have greater bulk density, so a larger γ-ray irradiation dose may be required to eliminate the bacteria completely.

Although the bulk density of the multivitamin premixes was small and the material was fluffy, elimination of microorganisms in vitamin complexes B and C required 9 kGy of irradiation, likely due to their composition. Vitamins contain numerous double bonds, meaning that they can significantly increase the energy absorption of γ-rays [36]. This might explain why the higher dose of γ-ray irradiation resulted in decreased content of vitamins B1 and C [36]. However, γ-ray irradiation had no effect on the vitamin A and E content in the samples, and this finding requires further investigation.

Another objective of our study was to investigate the effect of high bacteria loads and *E. coli* contamination, with or without γ-ray irradiation, on the feed value of fishmeal fed to weaning piglets. To exclude the effect of bacteria in the basal diet on the growth performance of piglets, this diet was irradiated with γ-rays to eliminate microorganisms. All irradiated feed used in this experiment was stored in sealed receptacles, and microbial counts were conducted after γ-ray treatment. However, the feed could have been contaminated by microorganisms from the environment during transportation, storage, and feeding. However, we ignored the impact of these microorganisms in the environment on the feeding value of the feeds provided to the piglets because the interval between the unpackaging of the feed and feeding was short.

We found that the piglets fed the diet containing 6% fish meal with a higher bacteria load and contaminated with *E. coli* had significantly lower average daily weight gain. This decreased growth performance might have been due in part to the lower feed intake (−58 g/d), although the difference was not statistically significant. The lower feed intake might be linked to the diarrhea caused by the presence of *E. coli* in the feed. Serum c-reactive protein, which is a biomarker of bacterial infection in swine [37], was present in the piglets fed the *E. coli*-contaminated fishmeal, but the level was same as that of the control group when the fishmeal was treated with γ-ray irradiation. The immature intestinal tract of weaning piglets is highly vulnerable to environmental factors [2,38], and pathogenic *E. coli* is one of the leading causes of post-weaning diarrhea [30,32,33]. Accordingly, we found that the number of viable *E. coli* counts in the feces was significantly increased when the weaning piglets consumed the diet containing *E. coli*-contaminated fishmeal. The number of viable *E. coli* counts in the feces is considered to be an important parameter of the gut health of weaning piglets [39], and our results showed that *E. coli*-contaminated fishmeal was harmful to the weaning piglets. 

γ-ray irradiation has been widely applied in various fields, including food sterilization [40], healthcare [41], and the packaging materials sector [42]. On the other hand, the cost of using γ-ray irradiation for sterilization falls within an acceptable range. For instance, irradiating fishmeal at a dose of 9 kGy only adds a modest 5.6% to the overall cost. Importantly, there is no potential radiation residue in the feed that could harm animals. The development of the civil nuclear power industry in China [43] is likely to stimulate the use of γ-ray irradiation in livestock production and the feed industry. However, there are concerns regarding the environmental safety of γ-ray irradiation. One primary concern is the potential harm to the environment due to ionizing radiation. Gamma rays, a form of ionizing radiation, have the ability to damage living organisms, including plants and animals. Therefore, it is critical to ensure the safe containment and control of the radiation source. Moreover, the use of gamma ray irradiation can generate radioactive waste, which must be managed safely. Proper storage, handling, and disposal of this waste are essential to prevent environmental contamination. Facilities employing gamma ray irradiation must have suitable shielding and containment structures in place to prevent the leakage of radiation into the environment. These facilities are subject to regulatory requirements to ensure their safety.

Nevertheless, our study revealed that weaning piglets fed diets containing pathogenic microorganisms had impaired gut function and growth performance. Similar to our results, DeRouchey et al. previously reported that irradiation improved the feed value of blood meal with respect to nursery pig performance [44]. Therefore, it is important to eliminate harmful bacteria in feed to protect weaning piglets from environmental stress. We found that the contamination of fishmeal with *E. coli* can be completely alleviated via γ-ray irradiation and that the negative effect of *E. coli*-contaminated fishmeal on weaning piglets can be totally reversed via γ-ray irradiation of the feed. These results suggest that γ-ray irradiation is sufficiently suitable and safe for attenuating biosafety concerns about feeds of animal origin. However, whether microorganisms other than *E. coli* that have non-pathogenic potential affect the growth performance and gut health of weaning piglets remains unknown.

## 5. Conclusions

Collectively, our results showed that high microbial load and *E. coli* contamination could induce impaired gut health and growth performance in weaning piglets. However, γ-ray irradiation at a dose of 6–9 kGy was sufficient to eliminate most of the microorganisms in the feed. Based on these findings, γ-ray irradiation can be used to attenuate the biosafety concerns regarding protein feed of animal origin for livestock animals.

## Figures and Tables

**Table 1:** Diet composition and nutrient content (%).

	Basal	Control	FM ^3^-Contaminated	FM-Irradiation
Corn	62.13	62.13	62.13	62.13
Soybean meal, CP46%	15.00	15.00	15.00	15.00
Extruded soybean	10.00	10.00	10.00	10.00
Fish meal, Sample C		6.00		
Fish meal, Sample A			6.00	
Fish meal, Sample A, irradiation				6.00
Soybean oil	2.00	2.00	2.00	2.00
Sucrose	2.00	2.00	2.00	2.00
*L*-Lys HCL, 98%	0.30	0.30	0.30	0.30
*DL*-Met, 98.5%	0.10	0.10	0.10	0.10
*L*-Thr, 98%	0.04	0.04	0.04	0.04
*L*-Trp, 98%	0.01	0.01	0.01	0.01
Choline chloride, 50%	0.15	0.15	0.15	0.15
Limestone	0.87	0.87	0.87	0.87
CaHPO_4_	0.65	0.65	0.65	0.65
NaCl	0.50	0.50	0.50	0.50
Mineral premix ^1^	0.20	0.20	0.20	0.20
Vitamin premix ^2^	0.05	0.05	0.05	0.05
Total	94.00	100.00	100.00	100.00

^1^ Supplied per kg of diet (mg/kg): Fe, 110.0 mg (FeSO_4_·7H_2_O); Cu, 6 mg (CuSO4·5H_2_O); Mn, 4.0 mg (MnSO_4_·H_2_O); Zn, 100 mg (ZnSO_4_); I, 0.15 mg (KI); Se, 0.30 mg (Na_2_SeO_3_). ^2^ Supplied per kg of diet (mg/kg): vitamin A (transretiny lacetate), 15,000 IU; vitamin D_3_ (cholecalciferol), 5000 IU; vitamin E (all-rac-tocopherol-acetate), 40 IU; vitamin K (bisulfate menadione complex), 35 mg; riboflavin, 5 mg; pantothenic acid (d-Capantothenate), 25 mg; nicotinic acid, 30 mg; pyridoxine (pyridoxine HCl), 5 mg; thiamine (thiamine mononitrate), 2 mg; vitamin B_12_ (cyanocobalamin), 0.03 mg; D-biotin, 0.25 mg; folic acid, 2.5 mg. ^3^ The FM-contaminated diet was supplemented with 6% FM with a high bacteria count and contaminated with *E. coli*. The irradiated FM diet was supplemented with 6% contaminated FM that had been treated with γ-ray irradiation.

**Table 2:** Analyzed nutrient content (%, as-fed).

	Control	FM ^1^-Contaminated	Irradiated FM
Estimated DE, Mcal/kg	3.52	3.52	3.52
Crude protein	18.98	19.04	19.05
Calcium	0.85	0.84	0.85
Phosphorus	0.63	0.65	0.64
Ether extract	6.73	6.71	6.69
Neutral detergent fiber	7.56	7.61	7.60
Ash	2.95	2.93	2.97
Lysine	1.32	1.35	1.34
Methionine	0.46	0.45	0.45
Cysteine	0.30	0.31	0.30
Threonine	0.81	0.83	0.84
Tryptophan	0.23	0.24	0.23
Valine	0.91	0.92	0.90
Arginine	1.24	1.24	1.23
Isoleucine	0.78	0.79	0.78
Leucine	1.65	1.66	1.67

^1^ FM, fishmeal. The FM-contaminated diet was supplemented with 6% FM with high bacteria count and contaminated with *E. coli*. The irradiated FM diet was supplemented with 6% contaminated FM that had been treated with γ-ray irradiation.

**Table 3:** Effect of γ-ray irradiation on the amino acid profiles of fishmeal samples *.

	Sample A					Sample B					Sample C				
γ-Rays, kGy	0	3	6	9	SEM	0	3	6	9	SEM	0	3	6	9	SEM
lysine	4.55	4.59	4.61	4.51	0.23	4.92	4.89	5.03	4.99	0.28	4.92	4.89	5.03	4.99	0.21
methionine	1.83	1.86	1.87	1.81	0.12	1.81	1.85	1.79	1.83	0.09	1.81	1.85	1.74	1.83	0.08
tryptophan	0.53	0.56	0.55	0.52	0.03	0.73	0.75	0.72	0.73	0.08	0.73	0.75	0.70	0.73	0.05
threonine	2.61	2.67	2.62	2.59	0.15	2.78	2.68	2.84	2.69	0.14	2.78	2.68	2.84	2.69	0.12
arginine	3.90	3.93	3.89	3.79	0.20	3.99	4.04	4.08	3.89	0.31	3.99	4.04	4.08	3.89	0.19
phenylalanine	2.44	2.41	2.49	2.51	0.19	2.64	2.70	2.54	2.61	0.34	2.64	2.70	2.54	2.61	0.11
leucine	4.53	4.52	4.58	2.49	0.23	4.64	4.75	4.47	4.61	0.24	4.64	4.75	4.47	4.61	0.29
isoleucine	2.63	2.63	2.64	2.59	0.13	2.51	2.57	2.42	2.58	0.23	2.51	2.57	2.42	2.58	0.11
valine	3.14	3.15	3.16	3.12	0.16	3.21	3.09	3.28	3.11	0.18	3.21	3.09	3.28	3.11	0.13
histidine	1.42	1.41	1.43	1.42	0.07	1.71	1.65	1.75	1.62	0.19	1.71	1.65	1.75	1.62	0.12
alanine	3.89	3.91	3.99	3.92	0.23	4.04	3.89	4.13	3.91	0.21	4.04	3.89	4.13	3.91	0.22
aspartic acid	5.12	5.22	5.24	5.23	0.27	5.38	5.50	5.18	5.37	0.28	5.38	5.50	5.18	5.37	0.23
cysteine	0.87	0.89	0.91	0.88	0.06	0.96	0.98	0.92	0.93	0.05	0.96	0.98	0.92	0.93	0.06
glutamic acid	7.93	7.99	7.87	7.57	0.46	8.20	8.39	7.90	7.98	0.42	8.20	8.39	7.90	7.98	0.26
glycine	4.93	4.99	4.83	4.82	0.25	5.14	5.26	4.95	5.04	0.37	5.14	5.26	4.95	5.04	0.21
proline	2.29	2.32	2.41	2.84	0.18	2.42	2.47	2.33	2.66	0.13	2.42	2.47	2.33	2.66	0.13
serine	2.21	2.23	2.31	2.34	0.16	2.33	2.38	2.24	2.36	0.18	2.33	2.38	2.24	2.36	0.17
tyrosine	1.82	1.88	1.87	1.91	0.17	1.97	2.01	1.98	1.96	0.14	1.97	2.01	1.98	1.96	0.09

* No statistical significance was observed for any fishmeal samples at each γ-ray irradiation dose.

**Table 4:** Effect of γ-ray irradiation on the content of some vitamins in the vitamin complex samples.

Sample	Vitamins	γ-Ray Irradiation Dose, kGy				SEM	*p* Value
		0	3	6	9		
Complex A	Vitamin A (g/kg)	18.5	18.2	17.3	17.2	1.23	>0.05
	Vitamin E (g/kg)	8.5	8.4	8.3	8.3	0.58	>0.05
	Vitamin B1 (g/kg)	12.3 ^a^	10.3 ^b^	9.4 ^bc^	7.6 ^c^	0.64	<0.05
	Vitamin C (g/kg)	220.2 ^a^	210.3 ^ab^	180.9 ^b^	160.5 ^bc^	12.87	<0.05
Complex B	Vitamin A (g/kg)	78.5	77.9	78.3	76.2	7.25	>0.05
	Vitamin E (g/kg)	14.8	13.8	13.1	12.8	1.27	>0.05
	Vitamin B1 (g/kg)	21.9 ^a^	17.8 ^ab^	16.8 ^b^	13.4 ^b^	1.63	<0.05
	Vitamin C (g/kg)	120.3 ^a^	110.4 ^ab^	104.9 ^ab^	92.6 ^b^	6.99	<0.05
Complex C	Vitamin A (g/kg)	25.8	24.9	24.7	24.8	2.34	>0.05
	Vitamin E (g/kg)	19.2	18.5	18.3	19.3	1.76	>0.05
	Vitamin B1 (g/kg)	14.9 ^a^	12.7 ^ab^	11.2 ^b^	8.9 ^bc^	1.11	< 0.05
	Vitamin C (g/kg)	n.d.	n.d.	n.d.	n.d.	-	-

Different letters (^a, b, c^) denote statistical significance at *p* < 0.05.

**Table 5:** Effect of γ-ray irradiated fishmeal on bacterial counts in the feeds.

	Basal Diet ^1^	Fish Meal Samples		
		^2^ Sample C	Sample A	Sample A + Irradiation ^2^
Bacteria counts, CFU/g	n.d.	40	91,500	n.d.
Presence of *E. coli*	–	–	+	–
TVB-N, mg/100 g	/	38	235	183

^1^ The basal diet and fishmeal sample A were treated with γ- irradiation at a dosage of 6 kGy. ^2^ Samples A and C were the same as those in Figure 1. TVB-N, total volatile basic nitrogen. +, positive; –, negative; /, not applicable.

**Table 6:** Effect of γ-ray irradiated fishmeal on the growth performance of weaning piglets ^1^.

		Control	FM-Contaminated	Irradiated FM	SEM	*p* Value
BW, kg	Day 1	6.97	6.98	6.98	0.15	>0.05
	Day 7	8.57	8.11	8.42	0.20	>0.05
	Day 14	10.37	9.48	10.29	0.29	>0.05
ADG, g/d	Days 1–7	227.5 ^a^	161.3 ^b^	206.2 ^a^	14.2	<0.05
	Days 8–14	257.7 ^a^	196.3 ^b^	267.6 ^a^	18.4	<0.05
	Days 1–14	242.6 ^a^	178.8 ^b^	236.9 ^a^	13.3	<0.05
ADFI, g/d	Days 1–7	348.3	273.4	329.2	22.2	>0.05
	Days 8–14	503.0	453.4	519.8	35.5	>0.05
	Days 1–14	424.4	366.5	424.5	25.1	>0.05
FCR	Days 1–7	1.55	1.78	1.60	0.10	>0.05
	Days 8–14	1.99	2.39	2.05	0.17	>0.05
	Days 1–14	1.77	2.08	1.82	0.10	>0.05

^1^ BW, bodyweight; ADG, average daily gain; ADFI, average daily feed intake; FCR, feed conversion ratio, calculated as the ratio of feed to gain. Different letters (^a, b^) denote statistical significance at *p* < 0.05.

**Table 7:** Effect of γ-ray irradiated fishmeal on the diarrhea index of weaning piglets ^1^.

	Control	FM-Contaminated	Irradiated FM	SEM	*p* Value
Days 1–7	1.19 ^b^	2.02 ^a^	1.17 ^b^	0.20	<0.05
Days 8–14	0.58 ^b^	1.32 ^a^	0.62 ^b^	0.12	<0.05
Days 1–14	0.89 ^b^	1.67 ^a^	0.90 ^b^	0.13	<0.05

^1^ FM, fishmeal. The FM-contaminated diet was supplemented with 6% FM with high bacteria count and contaminated with *E. coli*. The irradiated FM diet was supplemented with 6% contaminated FM with γ-ray irradiation at a dose of 6 kGy. Different letters (^a, b^) denote statistical significance at *p* < 0.05.

## Data Availability

The data in the present study can be made available upon reasonable request made to the corresponding author.
